# Factors associated with sleep state misperception in patients with obstructive sleep apnea: a cross-sectional study

**DOI:** 10.3389/fneur.2026.1925074

**Published:** 2026-09-01

**Authors:** Tianyu Jing, Mengjiao Xu, Yuping Wang, Yunyan Cai, Yu Gong, Liyun Zhu

**Affiliations:** 1 Department of Respiratory Medicine, Jiangyin Third People's Hospital, Jiangyin, China; 2 Department of Cardiology, Jiangyin People’s Hospital, Jiangyin, China; 3 Sleep Center, Jiangyin Third People's Hospital, Jiangyin, China

**Keywords:** comorbid insomnia and sleep apnea, DBAS-16, obstructive sleep apnea, PSG, sleep state misperception

## Abstract

**Objectives:**

This study aimed to investigate factors associated with sleep state misperception (SSM) in patients with obstructive sleep apnea (OSA), and to provide evidence for the early identification and intervention of comorbid insomnia and obstructive sleep apnea (COMISA).

**Methods:**

This cross-sectional study enrolled 700 patients diagnosed with OSA by polysomnography (PSG) at a sleep center of a hospital in Jiangsu Province, China, between June 2024 and May 2026. Based on the Sleep Perception Index (SPI), patients were classified into three groups: a negative misperception group (*n* = 118), a normal perception group (*n* = 521), and a positive misperception group (*n* = 61). On the day of PSG, participants completed the Pittsburgh Sleep Quality Index (PSQI), Hospital Anxiety and Depression Scale (HADS), Dysfunctional Beliefs and Attitudes about Sleep Scale-16 (DBAS-16), Epworth Sleepiness Scale (ESS), Subjective Cognitive Decline Questionnaire (SCD-Q9), and Montreal Cognitive Assessment (MoCA). On the morning after PSG, subjective estimates of the previous night’s sleep were collected. Factors associated with sleep state misperception were identified using multinomial logistic regression analysis.

**Results:**

In the multinomial logistic regression analysis with the normal perception group as the reference, higher N3 sleep stage percentage (N3%), higher apnea–hypopnea index (AHI), lower PSQI score, and lower sleep efficiency (SE) were independent factors associated with positive misperception (all *p* < 0.05). Higher AHI, lower SCD-Q9 score, lower DBAS-16 score, lower HADS-Anxiety (HADS-A) score, and lower SE were independent risk factors associated with negative misperception (all *p* < 0.05).

**Conclusion:**

The direction of sleep state misperception in patients with OSA is specifically associated with multidimensional clinical characteristics. High AHI was independently associated with both positive and negative misperception. Positive misperception was more closely linked to disruptions in objective sleep architecture and to better self-rated sleep quality, whereas protective factors against negative misperception involved more rational sleep-related beliefs, more subjective cognitive decline complaints, and higher levels of anxiety. This divergence suggests that different misperception directions may be related to distinct underlying psychopathological and neurocognitive mechanisms, underscoring the need for precision screening and stratified intervention strategies tailored to the direction of misperception in clinical practice.

## 1 Introduction

Sleep perception refers to an individual’s self-evaluation of their sleep state (1), encompassing both sleep duration and quality, and has become a primary basis for the diagnosis of sleep disorders and the evaluation of treatment efficacy (2). Comparisons between subjective reports and objective sleep parameters measured by polysomnography (PSG) have revealed a marked discrepancy in some patients with insomnia (3). This discrepancy has been termed sleep state misperception (SSM). In most instances, this misperception takes the form of negative misperception, whereby individuals substantially underestimate total sleep time (TST) relative to PSG-recorded values. In contrast, the overestimation of TST is referred to as positive sleep misperception (4). Patients with positive misperception, although they experience objective sleep deprivation, fail to perceive it, which may lead to delayed diagnosis and treatment. Conversely, patients with negative misperception have no objective insomnia, yet their subjective perception of insufficient sleep can readily trigger anxiety and result in adverse outcomes. This misperception has thus become a significant factor hindering accurate diagnosis and treatment evaluation of sleep disorders (5).

Obstructive sleep apnea (OSA) is characterized by recurrent nocturnal apnea and hypopnea, leading to severe intermittent hypoxia and disruption of sleep architecture (6). As a common chronic sleep-disordered breathing condition, OSA is associated with substantial interindividual variability in sleep state perception (7). OSA patients with negative misperception, often motivated by the perception of insufficient sleep, often make more vigorous attempts to fall asleep. According to the attention–intention–effort model of insomnia, such active attempts paradoxically may accelerate the objective progression of insomnia (8). Therefore, OSA patients with sleep state misperception may be more likely to develop comorbid insomnia over time, suggesting that SSM may constitute a prodromal or transitional phase preceding the onset of insomnia in patients with OSA (9).

In recent years, the co-occurrence of OSA and insomnia has drawn increasing attention, giving rise to the specialized term comorbid insomnia and obstructive sleep apnea (COMISA) (10). Patients with COMISA have a higher risk of cardiovascular and cerebrovascular complications, greater functional impairment, and poorer quality of life (11). Furthermore, insomnia and OSA can mutually exacerbate each other and complicate their respective treatment (12). Based on this, we speculate that in OSA patients without comorbid insomnia, SSM may serve as a valuable indicator for identifying those at risk of future progression to COMISA. Moreover, identifying factors associated with SSM may reveal therapeutic targets in OSA management, thereby offering new avenues for preventing COMISA. Therefore, recognizing and addressing SSM is of significant clinical importance in this population. However, previous studies have predominantly examined sleep misperception as a unidirectional phenomenon or have been limited by small sample sizes. Integrating comprehensive polysomnographic, neuropsychological, and questionnaire data from 700 patients with polysomnography-confirmed OSA, the present study used multidimensional analysis to explore distinct correlates of positive versus negative sleep state misperception, potentially guiding the early identification and stratified intervention for high-risk patients.

## 2 Methods

### 2.1 Study participants

This study enrolled 700 patients diagnosed with OSA by PSG at the sleep center of a general hospital in Jiangsu Province, China, from June 2024 to May 2026. The inclusion criteria were as follows: (1) Chinese adults aged ≥18 years; (2) diagnosis of OSA confirmed by PSG according to the 2018 Chinese guideline for the primary care of adult obstructive sleep apnea (13); (3) TST > 2 h; (4) no consciousness disturbance and able to communicate clearly; (5) voluntarily participating in the study. The exclusion criteria were: (1) presence of severe complications or comorbidities; (2) co-existing chronic conditions that may affect sleep perception and quality, including Parkinson’s disease, depression, anxiety disorder, cancer, chronic kidney disease, tinnitus, and chronic pain; (3) use of medications significantly affecting sleep or cognition; (4) concurrent other sleep disorders, including restless legs syndrome, rapid eye movement sleep behavior disorder, and narcolepsy.

This study was approved by the Ethics Committee of the Jiangyin Third People’s Hospital (approval No. SY2025-032-01) and was conducted in accordance with the principles of the Declaration of Helsinki. All patients and their families were informed of the study details and signed the informed consent form.

### 2.2 General information and measures

#### 2.2.1 General information

We collected demographic information from all participants, including height, weight, history of smoking and alcohol consumption, and history of chronic diseases. All participants completed sleep-related questionnaires and cognitive function assessments in a quiet, comfortable, and private room.

#### 2.2.2 Measures

Six instruments were used in this study. Permission for use was obtained from the original authors or authorized translators where required.

Daytime sleepiness was assessed using the Chinese version of the Epworth Sleepiness Scale (ESS) (14). The ESS is an 8-item scale designed to evaluate an individual’s likelihood of dozing off or falling asleep in eight common daily situations, with higher scores indicating more severe daytime sleepiness.

Anxiety and depressive symptoms were screened using the Hospital Anxiety and Depression Scale (HADS) (15). The HADS comprises two subscales, the Anxiety subscale (HADS-A) and the Depression subscale (HADS-D), each consisting of 7 items (14 items in total). Higher scores reflect more severe anxiety or depressive symptoms.

Subjective sleep quality over the past month was assessed using the Pittsburgh Sleep Quality Index (PSQI) (16). The PSQI evaluates seven domains: subjective sleep quality, sleep latency, total sleep time, sleep efficiency, sleep disturbances, use of sleep medication, and daytime dysfunction. Each domain is scored from 0 to 3, yielding a global score ranging from 0 to 21, with higher scores indicating poorer subjective sleep quality.

Objective and subjective cognitive function were assessed using the Chinese version of the Montreal Cognitive Assessment (MoCA) (17) and the Subjective Cognitive Decline Questionnaire (SCD-Q9) (18), respectively. The MoCA is a validated screening instrument for mild cognitive impairment, covering visuospatial and executive function, memory, attention and concentration, language, abstraction, calculation, and orientation. The total score is 30, with higher scores indicating better global cognitive function. To facilitate efficient and effective assessment in the local context, the Beijing version of the MoCA (19) was adopted in the present study. The SCD-Q9 is a single-dimension, self-report questionnaire designed to systematically evaluate subjectively perceived cognitive decline. It contains 9 items, with a total score ranging from 0 to 9, and higher scores indicate a greater number and frequency of subjective cognitive complaints.

Dysfunctional sleep-related beliefs and attitudes were assessed using the Chinese version of the Dysfunctional Beliefs and Attitudes about Sleep Scale-16 (DBAS-16) (20), which has been validated in a Chinese population by Fu et al. The DBAS-16 is a self-report questionnaire comprising 16 items across four domains: consequences of insomnia, worry about sleep, sleep expectations, and medication dependency. Items are rated on a 5-point Likert scale, and the total score ranges from 16 to 80. Lower scores reflect more dysfunctional beliefs and attitudes about sleep.

#### 2.2.3 PSG and morning questionnaire

All participants underwent overnight PSG (>7 h) in a light-attenuated, sound-controlled sleep laboratory using a SOMNOmedics GmbH system. Alcohol, caffeine, and stimulant/sedative medications were prohibited on the day of monitoring. Electroencephalogram (EEG), mandibular electromyography (EMG), electrooculography (EOG), nasal airflow, oro-nasal thermistor transducer, thoracic and abdominal movements, electrocardiogram, body position, oxygen saturation, snoring, video, and other signals were recorded continuously throughout the night. The division of each sleep stage and sleep-related respiratory events was manually interpreted by a specialized sleep physician based on the standards of the American Academy of Sleep Medicine (AASM) (21). Nocturnal sleep related parameters, including TST, sleep efficiency (SE), sleep onset latency (SOL), REM latency, wake after sleep onset (WASO), non-rapid eye movement stage 1 percentage (N1%), non-rapid eye movement stage 2 percentage (N2%), non-rapid eye movement stage 3 percentage (N3%), rapid eye movement stage percentage (REM%), AHI, REM-AHI, NREM-AHI, oxygen desaturation index (ODI), arousal index (ArI), mean oxyhemoglobin saturation (mean SaO_2_), lowest oxyhemoglobin saturation (lowest SpO_2_), respiratory event-related arousal index (RERAI), N2 spindle index, N3 spindle index, and periodic limb movement index (PLMI), were continuously collected from the subjects.

The sleep spindle related parameters were derived mainly from the central EEG (C4: M1), and the sleep spindle features were extracted as independent signals according to the algorithm established by Molle et al. (22), which used the Fourier transform to convert the EEG data into the frequency domain; then, 11–16 Hz bandpass filtering was performed. After that, signal smoothing was performed using a moving average method in 200 ms units, and finally, feature selection was performed on the basis of duration and amplitude (peak-to-trough amplitude).

On the morning following the PSG, all participants were required to complete a morning questionnaire to record their subjective estimates of TST, SOL, and WASO, as well as a subjective rating of sleep quality (options: “very good,” “good,” “poor,” “very poor”). The questionnaire was administered within 30 min after the completion of PSG.

#### 2.2.4 Relative discrepancy between subjective and objective sleep parameters

To date, no universally accepted definition or cut-off value exists for sleep misperception, the sleep perception index (SPI). The SPI defined as the ratio of subjective total sleep time (sTST) to objective total sleep time (oTST) recorded by PSG, is one of the most commonly used measures of sleep perception (23, 24). An SPI closer to 100% indicates greater concordance between sTST and oTST; SPI < 100% indicates underestimation of TST, and SPI > 100% indicates overestimation of TST. Drawing on a recently published study investigating factors associated with sleep perception in OSA patients (25), the present study categorized SPI into three groups: a negative misperception group (SPI < 80%), a normal perception group (80% ≤ SPI ≤ 120%), and a positive misperception group (SPI > 120%). It should be noted that although such 80%/120% thresholds have been adopted in studies on sleep perception in OSA (26), their applicability has not been specifically validated and requires further investigation in future studies.

### 2.3 Data analysis

All data were analyzed using SPSS Statistics (version 27.0; IBM Corp., Armonk, NY, USA). Continuous variables were assessed for normality using the Shapiro–Wilk test. Normally distributed data were presented as mean ± standard deviation (SD), while non-normally distributed data were expressed as median (interquartile range [IQR]; Q1–Q3). Between-group differences in continuous variables were compared using one-way analysis of variance (ANOVA), and categorical variables were compared using the chi-square test. *Post hoc* pairwise comparisons were performed with the Bonferroni correction, using the normal perception group as the reference. Variables that showed significant differences in between-group comparisons were entered as candidates into a multinomial logistic regression model to identify factors associated with sleep state misperception. A two-tailed *p* value < 0.05 was considered statistically significant. Model performance for the multinomial logistic regression was evaluated using the likelihood ratio test, Nagelkerke’s pseudo-*R*^2^, and the deviance goodness-of-fit test.

## 3 Results

### 3.1 Baseline characteristics, sleep-related questionnaires, and cognitive function scores

**Figure 1:**
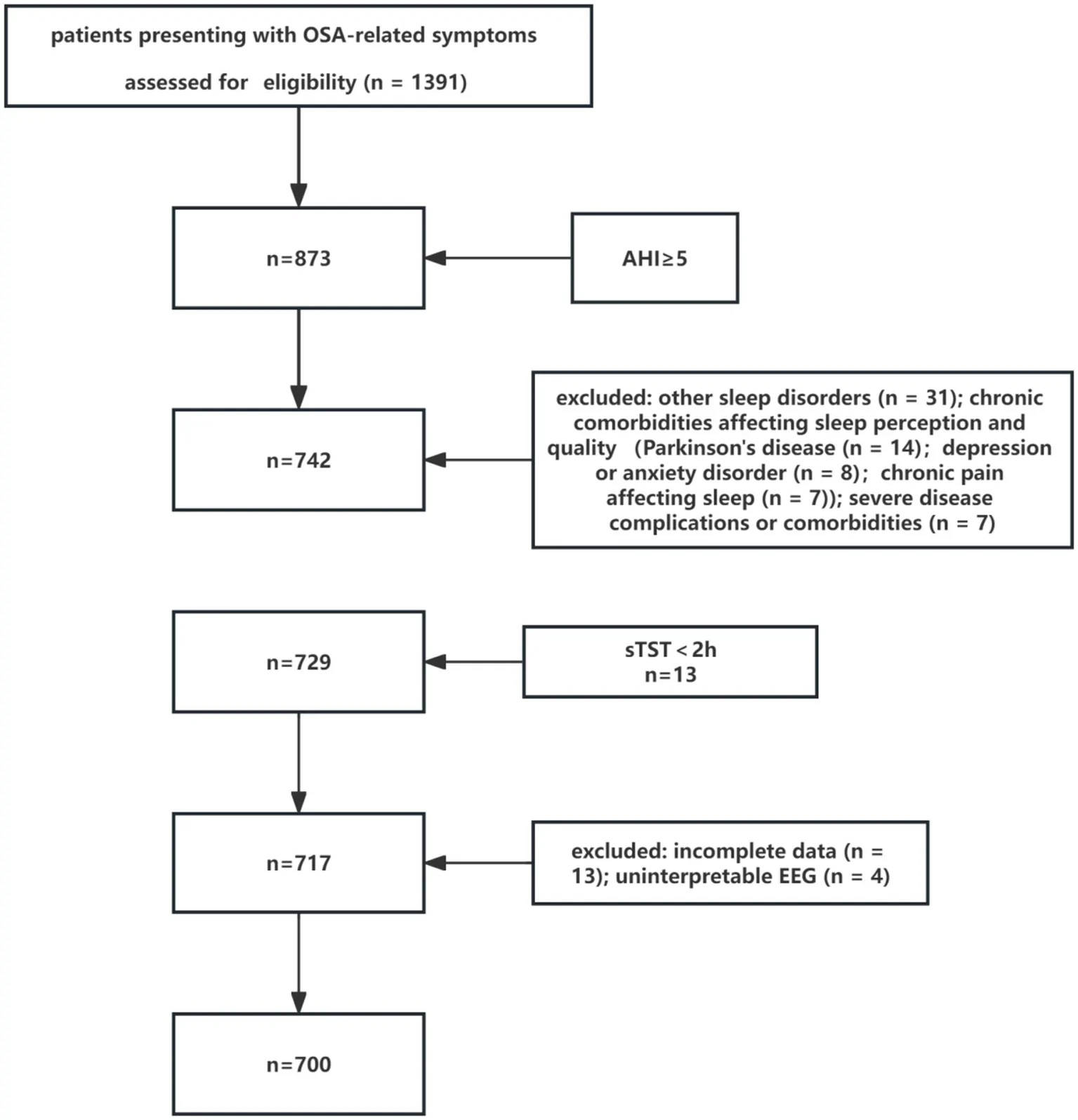
Flow chart.

A total of 700 patients who met the inclusion and exclusion criteria and completed all subjective and objective assessments were enrolled (the screening flow chart is shown in Figure 1). Based on SPI, participants were classified into three groups: a positive misperception group (*n* = 61), a normal perception group (*n* = 521), and a negative misperception group (*n* = 118). Notably, the positive misperception group was substantially smaller than the negative misperception and normal perception groups. This imbalance may reduce statistical power and the stability of regression estimates specific to this group. Therefore, corresponding findings should be interpreted with caution. The demographic characteristics, sleep-related questionnaire scores, and cognitive function scores of each group are presented in Table 1. No statistically significant differences were observed among the three groups in age, BMI, smoking history, alcohol consumption history, hypertension, diabetes mellitus, arrhythmia, coronary heart disease, daytime sleepiness, depressive symptoms, or objective cognitive function (all *p* > 0.05).

**Table 1:** Baseline demographic characteristics and questionnaire scores of the study participants.

Characteristics	Positive misperception group *n* = 61 (8.7%)	Normal perception group *n* = 521 (74.4%)	Negative misperception group *n* = 118 (16.9%)	*p* value
Male, *n* (%)	48 (78.7%)^Δ^	332 (63.7%)	72 (61.0%)^Δ^	0.046
Age, years	61.27 ± 15.48	61.12 ± 13.32	61.85 ± 14.56	0.842
BMI, kg/m^2^	26.58 ± 3.81	25.76 ± 3.47	26.33 ± 4.36	0.104
Smoking, *n* (%)	29 (47.5%)	341 (65.5%)	80 (67.8%)	0.055
Drinking, *n* (%)	22 (36.1%)	184 (35.3%)	40 (33.9%)	0.573
Hypertension, *n* (%)	17 (27.9%)	171 (32.8%)	32 (27.1%)	0.398
Diabetes status, *n* (%)	7 (11.4%)	49 (9.4%)	11 (9.3%)	0.338
Arrhythmia, *n* (%)	3 (4.9%)	38 (7.3%)	12 (10.2%)	0.413
CHD, *n* (%)	9 (14.8%)	58 (11.1%)	17 (14.4%)	0.483
CI, *n* (%)	29 (47.5%)^*^	104 (31.5%)^*^	37 (31.4%)	0.038
ESS	6 (3, 10)	6 (3, 11)	6 (2, 10)	0.541
PSQI	6 (5, 8)^Δ,*^	8 (5, 12)^*,#^	12 (8, 15)^Δ,#^	<0.001
HADS (A)	1 (0, 3)^Δ^	1 (0, 3)^#^	2 (0, 4)^#,Δ^	0.041
HADS (D)	1 (0, 3)	1 (0, 3)	1 (0, 4)	0.466
SCD-Q9	3.5 (1.5, 5.5)^Δ^	4.5 (2, 6)	5 (3, 6.5)^Δ^	0.031
DBAS-16	44 (42, 47)^Δ,*^	43 (39, 46)^*,#^	38 (34, 42)^Δ,#^	<0.001
MoCA	20.52 ± 5.68	19.34 ± 6.33	19.16 ± 6.56	0.34

Continuous variables were compared using one-way analysis of variance or non-parametric tests, and categorical variables using the chi-square test, with Bonferroni correction applied for post-hoc comparisons.

^#^Negative misperception group vs. Normal perception group, *p* < 0.05.

^Δ^Negative misperception group vs. Positive misperception group, *p* < 0.05.

^*^Normal perception group vs. Positive misperception group, *p* < 0.05.

BMI, body mass index; CHD, coronary heart disease; CI, cerebral infarction; ESS, Epworth Sleepiness Scale; PSQI, Pittsburgh Sleep Quality Index; HADS-A, Hospital Anxiety and Depression Scale-Anxiety subscale; HADS-D, Hospital Anxiety and Depression Scale-Depression subscale; SCD-Q9, Subjective Cognitive Decline Questionnaire-9; MoCA, Montreal Cognitive Assessment; DBAS-16, Dysfunctional Beliefs and Attitudes about Sleep Scale-16.

In terms of sleep-related questionnaire scores, the negative misperception group had the highest PSQI score and the positive misperception group the lowest. The DBAS-16 score also showed significant intergroup differences, the negative misperception group scored significantly lower than both the normal perception group and the positive misperception group, indicating that patients in the negative misperception group held more dysfunctional sleep-related beliefs. Anxiety symptoms were significantly more severe in the negative misperception group than in the other two groups. The SCD-Q9 score was highest in the negative misperception group and lowest in the positive misperception group. In addition, the proportion of male patients was significantly higher in the positive misperception group than in the negative misperception group, and the prevalence of a history of cerebral infarction was significantly higher in the positive misperception group than in the normal perception and negative misperception groups.

### 3.2 Comparison of PSG characteristics among the three groups

Table 2 presents the sleep parameters. Significant differences were observed among the three groups in TST, SE, and WASO (all *p* < 0.01). The positive misperception group had the lowest TST and SE, significantly lower than those in the other groups (both *p* < 0.01), while WASO was significantly longer in the negative misperception group than in the other groups (*p* = 0.002). No significant differences were found in SOL or REM latency among the three groups (all *p* > 0.05). In terms of sleep architecture, N3% showed a significant intergroup difference (*p* = 0.002), with the negative misperception group having the lowest value, significantly lower than that in the normal perception group. No significant differences were observed in N1%, N2%, or REM% among the groups (all *p* > 0.05). Regarding respiratory events, the positive misperception group exhibited the greatest disease severity. AHI was significantly higher in this group than in the other groups (*p* < 0.001). REM-AHI, NREM-AHI, and ODI all showed the same distribution pattern across the three groups, with values being the highest in the positive misperception group, followed by the negative misperception group, and lowest in the normal perception group (all *p* < 0.001). RERAI was highest in the positive misperception group and significantly higher than in the other two groups (*p* < 0.001). In addition, the lowest SpO₂ in the positive misperception group and the mean SaO₂ in the negative misperception group were both significantly lower than those in the normal perception group (both *p* < 0.05). For neuroelectrophysiological markers, the N3 spindle index was lowest in the positive misperception group and significantly lower than that in the normal perception group (*p* = 0.035). No significant difference was found in the N2 spindle index among the three groups (*p* = 0.136).

**Table 2:** Polysomnographic characteristics of the study participants.

Characteristics	Positive misperception group *n* = 61 (8.7%)	Normal perception group *n* = 521 (74.4%)	Negative misperception group *n* = 118 (16.9%)	*p* value
TST (min)	351.61 ± 127.74^*^	425.22 ± 93.83^*,#^	389.76 ± 105.60^#^	<0.001
SE (%)	77.12 ± 14.99^*^	81.96 ± 11.92^*,#^	85.41 ± 15.53^#^	<0.001
SOL (min)	8 (2.7, 19.2)	6.6 (3.03, 13.45)	7.75 (3.08, 22.23)	0.150
REM latency (min)	80 (34.25, 147.25)	93 (55.25, 147.75)	75.75 (44.38, 144.5)	0.288
WASO (min)	66.7 (31.25, 133.5)	60.65 (32.36, 105.45)^#^	89.8 (44.85, 151.66)^#^	0.002
N1%	4.6 (2.1, 7.8)	4.55 (2.4, 8.8)	4.6 (2.58, 9.9)	0.628
N2%	63.5 (53.6, 70.55)	60.6 (51, 68.9)	62.6 (49.65, 74.5)	0.159
N3%	14.4 (3, 30.2)	15.5 (8.53, 24.1)^#^	10.7 (2.75, 18.55)^#^	0.002
REM%	15.4 (9.2, 19.4)	16.1 (10.93, 21.9)	15.4 (9.2, 19.4)	0.281
AHI (events/h)	40.7 (20.2, 63.1)^Δ,*^	19.85 (11.4, 35.08)^*,#^	26.7 (12.9, 43.35)^Δ,#^	<0.001
REM-AHI (events/h)	20.9 (7.25, 37.75)^*,Δ^	10.45 (2.35, 22.2)^*^	11.85 (2.48, 28.86)^Δ^	<0.001
NREM-AHI (events/h)	17.3 (7.85, 42.1)^Δ,*^	7.2 (2.23, 17.38)^*,#^	9.75 (3.58, 20.65)^Δ,#^	<0.001
ODI (events/h)	39.5 (17.55, 60.7)^Δ,*^	19.5 (11.1, 35.23)^*,#^	25.8 (12.38, 45.7)^*,#^	<0.001
ArI (events/h)	39.2 (20.4, 65.95)	31.9 (21, 44.78)^*^	36.55 (24.15, 62.58)^*^	0.006
Mean SpO_2_ (%)	93.81 ± 2.67	94.41 ± 2.58^#^	93.65 ± 3.60^#^	0.014
Lowest SpO_2_ (%)	80.56 ± 9.78^*^	83.41 ± 8.02^*^	82.64 ± 8.73	0.035
RERAI (events/h)	9.5 (2.8, 19.65)^*^	4.1 (2, 8.58)^*,#^	5.95 (2.38, 12.03)^#^	<0.001
N2 spindle index	116.1 (88.6, 187.9)	147.8 (92.4, 217.78)	129.7 (66, 228.13)	0.136
N3 spindle index	86.2 (53.3, 168.2)^*^	127.05 (64.15, 211.88)^*^	100.2 (53.63, 210.55)	0.035
PLMI (events/h)	7.3 (2, 24.55)	9.9 (2.8, 27.28)	11.1 (1.9, 25.8)	0.680

^#^Negative misperception group vs. Normal perception group, *p* < 0.05.

^Δ^Negative misperception group vs. Positive misperception group, *p* < 0.05.

^*^Normal perception group vs. Positive misperception group, *p* < 0.05.

TST, total sleep time; SE, sleep efficiency; SOL, sleep onset latency; WASO, wake after sleep onset; N1%, N1 sleep stage percentage; N2%, N2 sleep stage percentage; N3%, N3 sleep stage percentage; REM%, rapid eye movement sleep stage percentage; AHI, apnea–hypopnea index; REM-AHI, REM apnea–hypopnea index; NREM-AHI, NREM apnea–hypopnea index; ODI, oxygen desaturation index; ArI, arousal index; SpO₂, pulse oxygen saturation; RERAI, respiratory event-related arousal index; PLMI, periodic limb movement index.

### 3.3 Comparison of subjective and objective sleep parameters among the three groups

The positive misperception group had the shortest oTST but the longest sTST, demonstrating a marked overestimation of sleep duration. In contrast, the negative misperception group showed the shortest sTST, while the normal perception group exhibited roughly concordant subjective and objective sleep durations. Subjective sleep onset latency (sSOL) and objective wake after sleep onset (oWASO) were significantly higher in the negative misperception group than in the other two groups, with the negative misperception group also having the longest oWASO. Significant differences among the three groups were found for oWASO, subjective WASO (sWASO), and the WASO discrepancy (sWASO–oWASO) (Table 3).

**Table 3:** Sleep perception characteristics of the participants.

Characteristics	Positive misperception group *n* = 61 (8.7%)	Normal perception group *n* = 521 (74.4%)	Negative misperception group *n* = 118 (16.9%)	*p* value
oTST (min)	351.6 ± 127.74^*^	425.22 ± 93.83^*,#^	389.7 ± 105.60^#^	<0.001
STST, (min)	436.2 ± 147.28^*^	409.49 ± 98.31^*,#^	295.83 ± 87.26^#^	<0.001
TSTdis, (min)	84.61 ± 24.46^Δ,*^	−15.74 ± 37.16^*,#^	−93.94 ± 27.39^Δ,#^	<0.001
oSOL, (min)	8 (2.7, 19.2)	6.6 (3.03, 13.45)	7.75 (3.08, 22.23)	0.288
sSOL, (min)	15 (5, 30)^Δ^	15 (10, 30)^#^	30 (15, 45)^#,Δ^	<0.001
SOLdis, (min)	4.2 (0.15, 10.8)^Δ,*^	6.5 (2.95, 15.6)^*,#^	15.4 (5.23, 25.4)^Δ,#^	<0.001
oWASO, (min)	66.7 (31.25, 133.5)	60.65 (32.36, 105.45)^#^	89.8 (44.85, 151.66)^#^	0.002
sWASO, (min)	40 (2.0, 62.5)^Δ,*^	60 (3.75, 120)^*,#^	120 (60, 150)^Δ,#^	<0.001
WASOdis, (min)	−22.9 (−42.5, −0.55)^Δ,*^	3.3 (−12.35, 17.35)^*,#^	29 (6.7, 41.1)^Δ,#^	<0.001

^#^Negative misperception group vs. Normal perception group, *p* < 0.05.

^Δ^Negative misperception group vs. Positive misperception group, *p* < 0.05.

^*^Normal perception group vs. Positive misperception group, *p* < 0.05.

oTST, objective total sleep time; sTST, subjective total sleep time; TSTdis, discrepancy between sTST and oTST (sTST–oTST); oSOL, objective sleep onset latency; sSOL, subjective sleep onset latency; SOLdis, discrepancy between sSOL and oSOL (sSOL–oSOL); oWASO, objective wake after sleep onset; sWASO, subjective wake after sleep onset; WASOdis, discrepancy between sWASO and oWASO (sWASO–oWASO).

These findings indicate that the direction and magnitude of sleep perception bias were consistent across the three dimensions of TST, SOL, and WASO. The negative misperception group was characterized by a systematic overestimation of both SOL and WASO, whereas the positive misperception group exhibited a systematic underestimation of WASO.

### 3.4 Multinomial logistic regression analysis to identify factors associated with SSM

Based on significant differences observed in univariate analyses, combined with clinical relevance and prior literature, 11 variables were ultimately entered into the multinomial logistic regression model. The three misperception groups were set as the dependent variable, with the normal perception group serving as the reference category. The included variables were sex, history of cerebral infarction, PSQI, SCD-Q9, N3%, AHI, DBAS-16, HADS-A, sleep efficiency, mean SaO₂, and N3 spindle index. The overall model likelihood ratio test was significant (*χ*^2^ = 213.67, df = 22, *p* < 0.001), with a Nagelkerke pseudo-*R*^2^ of 0.342. Detailed regression results are presented in Table 4. It should be noted that the positive misperception group contained only 61 observations, falling below the generally recommended threshold of at least 10 events per variable (EPV ≥ 10). Consequently, the stability of parameter estimates for this group may be lower than for the other groups, and the confidence intervals may be wider. Findings for this group should therefore be interpreted with caution, and this limitation is further addressed in the Discussion.

**Table 4:** Multinomial logistic regression analysis of factors associated with sleep state misperception.

Variable	*β*	SE	*p*	OR (95%CI)
Positive misperception vs. Normal perception				
SCD-Q9	0.047	0.084	0.578	1.048 (0.89, 1.234)
PSQI	−0.123	0.054	0.024	0.885 (0.795, 0.984)
N3%	0.023	0.008	0.004	1.024 (1.008, 1.04)
AHI	0.047	0.008	<0.001	1.049 (1.033, 1.065)
DBAS-16	0.047	0.071	0.511	1.048 (0.912, 1.205)
HADS (A)	−0.029	0.08	0.718	0.971 (0.83, 1.137)
SE (%)	−0.032	0.011	0.004	0.968 (0.948, 0.99)
Mean SaO₂ (%)	0.035	0.061	0.572	1.035 (0.918, 1.167)
N3 spindle index	−0.002	0.001	0.057	0.998 (0.995, 1)
[SEX = Fe]	−0.288	0.359	0.422	0.75 (0.371, 1.514)
[SEX = M]	ref.			
[CI = Absent]	0.036	0.318	0.909	1.037 (0.556, 1.934)
[CI=Present]	ref.			
Negative misperception group vs. Normal perception group				
SCD-Q9	−0.153	0.058	0.008	0.858 (0.766, 0.961)
PSQI	0.053	0.036	0.141	1.054 (0.982, 1.131)
N3%	−0.001	0.008	0.93	0.999 (0.985, 1.014)
AHI	0.022	0.007	0.001	1.022 (1.009, 1.036)
DBAS-16	−0.227	0.039	0.001	0.797 (0.738, 0.861)
HADS (A)	−0.168	0.05	0.001	0.845 (0.767, 0.931)
SE (%)	−0.036	0.008	< 0.001	0.965 (0.949, 0.981)
Mean SaO₂ (%)	−0.042	0.038	0.266	0.959 (0.89, 1.033)
N3 spindle index	−0.001	0.001	0.337	0.999 (0.998, 1.001)
[SEX = Fe]	−0.054	0.245	0.825	0.947 (0.586, 1.533)
[SEX = M]	ref.			
[CI = Absent]	0.145	0.25	0.562	1.156 (0.708, 1.888)
[CI=Present]	ref.			

The reference category for the dependent variable is the normal perception group. Ref., reference group. All variance inflation factors (VIFs) were < 5, indicating no significant multicollinearity.

Fe, female; M, male; ref., reference. SCD-Q9, Subjective Cognitive Decline Questionnaire-9; PSQI, Pittsburgh Sleep Quality Index; N3%, N3 sleep stage percentage; AHI, apnea–hypopnea index; DBAS-16, Dysfunctional Beliefs and Attitudes about Sleep Scale-16; HADS-A, Hospital Anxiety and Depression Scale-Anxiety subscale; SE, sleep efficiency; SaO₂, arterial oxygen saturation; CI, cerebral infarction; OR, odds ratio; CI, confidence interval.

#### 3.4.1 Positive misperception group versus normal perception group

Higher N3% (OR = 1.024, 95% CI 1.008–1.040) and higher AHI (OR = 1.049, 95% CI 1.033–1.065) were independent associated with positive misperception. Higher PSQI score (OR = 0.885, 95% CI 0.795–0.984) and higher SE (OR = 0.968, 95% CI 0.948–0.990) were associated with a reduced risk of positive misperception. SCD-Q9, DBAS-16, HADS-A, mean SaO₂, N3 spindle index, sex, and history of cerebral infarction were not statistically significant in this comparison (Table 4).

#### 3.4.2 Negative misperception group versus normal perception group

Higher AHI (OR = 1.022, 95% CI 1.009–1.036) was an independent risk factor for negative misperception. Higher SCD-Q9 score (OR = 0.858, 95% CI 0.766–0.961), higher DBAS-16 score (OR = 0.797, 95% CI 0.738–0.861), higher HADS-A score (OR = 0.845, 95% CI 0.767–0.931), and higher SE (OR = 0.965, 95% CI 0.949–0.981) were all associated with a reduced risk of negative misperception. In other words, patients with more subjective cognitive decline, more rational sleep-related beliefs, higher levels of anxiety, and higher sleep efficiency were less likely to develop negative sleep misperception. N3%, mean SaO₂, N3 spindle index, sex, and history of cerebral infarction were not statistically significant in this comparison (Table 4).

## 4 Discussion

For OSA patients with SSM, accurate self-assessment of their condition is compromised, which may in turn be associated with reduced treatment adherence and inappropriate healthcare utilization (27). Previous studies have typically conceptualized SSM as a unidimensional phenomenon of either underestimation or overestimation and, limited by small sample sizes, have not systematically investigated how multidimensional factors are collectively associated with the direction of misperception (28, 29). The heterogeneity of sleep state misperception may reflect the interplay of multiple dimensions, including dysfunctional sleep-related beliefs, impaired emotion regulation, and sleep architecture disruption, posing new challenges for clinical intervention. Based on data from 700 patients with PSG-confirmed OSA, this study systematically analyzed multidimensional factors associated with the direction of misperception. The results suggested that AHI was a common independent correlate of both positive and negative misperception, indicating that greater OSA severity is associated with a higher likelihood of sleep perception deviating from normal, while the specific direction may be related to other accompanying factors. This finding extends previous knowledge on the simple association between AHI and sleep perception bias (26). Some patients with severe OSA lack a clear perception of their nocturnal apneas and arousals, possibly because long-term intermittent hypoxia may be linked to impaired memory encoding or interoceptive function, which could be associated with difficulty in accurately recalling sleep disruptions during the night (30). This may help explain the generally higher AHI observed in patients with positive misperception.

In our study, the association between higher N3% and positive misperception suggests that N3 sleep may be associated with a strong sense of sleep restoration (31), and this may be linked to patients’ ability to maintain a relatively positive appraisal of their sleep quality even when overall sleep architecture has been compromised by respiratory events. This interpretation is corroborated by the lower PSQI scores observed in the positive misperception group, further indicating that these patients subjectively perceived their sleep quality as acceptable.

The distribution of sleep perception bias exhibited marked group differences, most notably reflected in the significantly higher PSQI scores in the negative misperception group than in the positive misperception group, suggesting that negative misperception may be associated with catastrophic cognitions regarding sleep disturbance. Previous evidence indicates that patients with negative misperception tend to be excessively vigilant about their sleep quality and display elevated anxiety levels (32). At the neural level, such cognitive distortions may be related to dysfunction in the prefrontal cortex and amygdala. The former is involved in emotion regulation and decision-making, whereas the latter is involved in threat perception and response (33). Aberrant prefrontal activity may be associated with distorted subjective evaluation of sleep quality, which in turn disrupts emotional states and cognitive-behavioral patterns, potentially contributing to a vicious cycle (34).

In the present study, however, patients in the negative misperception group exhibited lower rather than higher anxiety levels, a finding that contrasts with traditional views and some prior evidence (33, 35). When interpreting this result, it should be noted that the exclusion of patients with formal anxiety or depressive disorders may have restricted the range of HADS-A scores, limiting the generalizability of this finding. The negative misperception group reported more subjective cognitive complaints and showed a trend toward lower MoCA scores, although the latter did not reach statistical significance. This pattern raises the possibility that some awareness of cognitive changes may coexist with distorted retrospective sleep appraisals rather than correcting them, contributing to sleep underestimation. One interpretation is that lower anxiety levels may co-occur with subtle cognitive dysfunction, rather than reflecting genuine emotional stability (36). Furthermore, this group also showed more dysfunctional sleep-related beliefs, which further suggests that cognitive distortions may play a role in sleep perception. Recent evidence has suggested that emotion regulation strategies mediate the relationship between sleep quality and psychological distress in patients with OSA (37). In addition, a range of demographic and psychosocial factors have been associated with poorer sleep quality and more sleep-related complaints in OSA populations, and may interact with cognitive and emotional processes to influence sleep perception (38). Collectively, these findings highlight the complexity of sleep perception in patients with OSA. Given the cross-sectional design of this study, no causal inferences can be drawn. Future longitudinal studies incorporating assessments of emotion regulation, psychological resilience, and neurocognitive function are warranted to further explore the underlying mechanisms.

Taken together, these findings suggest that SSM in OSA is not a unidimensional deviation but appears to results from the interplay among objective respiratory disturbances, sleep architecture alterations, psychological characteristics, and cognitive function. Future studies should aim to develop a clinically practical predictive model. For patients with positive misperception, such a model would alert clinicians that subjective comfort may mask severe OSA. In these cases, clinical decisions may be guided more by objective PSG metrics and supplemented by health education to correct underestimation of disease severity. Conversely, for patients with negative misperception, the PSG report could recommend cognitive-behavioral interventions targeting dysfunctional sleep beliefs and subjective cognitive complaints, combined with psychoeducation to alleviate excessively negative sleep appraisals. Such an approach would allow targeted interventions to be initiated before the comorbidity becomes fully established, potentially reducing adverse clinical outcomes. Furthermore, the observed link between subclinical anxiety and misperception suggests that incorporating brief psychological screening into routine OSA assessment may be valuable even without a formal anxiety diagnosis. These strategies may promote a more precise and individualized approach to OSA management, with care tailored to each patient’s misperception profile.

## 5 Limitations

This study has several limitations. First, the sample was recruited from a single sleep center, and all PSG recordings were acquired using a single system and scoring protocol. This may introduce selection bias and limit the generalizability of the findings to OSA patients in different clinical settings or geographic regions. Second, although the overall sample size was relatively adequate, the positive misperception group was small, which may lead to unstable odds ratio estimates and wide confidence intervals, particularly for comparisons involving this group. Therefore, these estimates should be interpreted with caution, and further validation in a larger sample is warranted. Third, the cross-sectional design precludes causal inference and only allows for the identification of associations. Fourth, patients with a formal clinical diagnosis of anxiety or depressive disorder were excluded, which may have restricted the range of HADS-A scores and thereby limited the generalizability of the observed psychological associations to the broader OSA population. Fifth, information on treatment status at the time of assessment was not available and could represent a potential confounder of sleep perception. Sixth, no consensus threshold exists for sleep misperception. We adopted the 80%/120% SPI cut-offs from a prior OSA study; however, these were originally derived from insomnia research and have not been validated in an OSA population. Findings based on this classification should therefore be interpreted with caution. Moreover, because SE and the denominator of the SPI both derive from objective total sleep time, a conceptual logical dependency exists between them that is not captured by standard collinearity diagnostics such as the variance inflation factor; therefore, the association between SE and sleep misperception should be regarded as preliminary. Finally, variables entered into the multivariable model were selected based on univariate analyses, a strategy that may introduce selection bias and could omit clinically relevant confounders. Consequently, the observed associations should be confirmed in future multicenter studies with larger, prespecified samples and validated assessment protocols.

## 6 Conclusion

In this large OSA sample, bidirectional heterogeneity of SSM was observed. AHI emerged as a common correlate of perceptual deviation, while dysfunctional sleep-related beliefs, anxiety levels, and N3% showed direction-specific associations with positive versus negative misperception. Future multicenter longitudinal studies integrating objective cognitive and neuroimaging assessments are needed to further explore the causal pathways and neural mechanisms involved. Such investigations may contribute to the development of targeted cognitive-behavioral interventions that could ultimately improve treatment adherence and subjective outcomes in patients with OSA.

## Data Availability

The raw data supporting the conclusions of this article will be made available by the authors, without undue reservation.

## References

[1] Walton TF Ree MJ Fueggle SN Bucks RS. A scoping review of sleep discrepancy methodology: what are we measuring and what does it mean? Sleep Med. (2025) 126:32–66. doi: 10.1016/j.sleep.2024.11.016, 39626529

[2] Li H Chen J Qi M Wang L Zhou S Chen J . Sleep architecture differences and predictive markers of sleep perception impairment in depression. Sci Rep. (2026) 16. doi: 10.1038/s41598-026-53826-4, 42168309 PMC13396371

[3] Liguori C Mombelli S Fernandes M Zucconi M Plazzi G Ferini-Strambi L . The evolving role of quantitative actigraphy in clinical sleep medicine. Sleep Med Rev. (2023) 68:101762. doi: 10.1016/j.smrv.2023.101762, 36773596

[4] Perrault AA Pomares FB Smith D Cross NE Gong K Maltezos A . Effects of cognitive behavioral therapy for insomnia on subjective and objective measures of sleep and cognition. Sleep Med. (2022) 97:13–26. doi: 10.1016/j.sleep.2022.05.010, 35691208

[5] Stephan AM Siclari F. Reconsidering sleep perception in insomnia: from misperception to mismeasurement. J Sleep Res. (2023) 32:e14028. doi: 10.1111/jsr.14028, 37678561

[6] Mohamed B Yarlagadda K Self Z Simon A Rigueiro F Sohooli M . Obstructive sleep apnea and stroke: determining the mechanisms behind their association and treatment options. Transl Stroke Res. (2024) 15:239–332. doi: 10.1007/s12975-023-01123-x, 36922470

[7] Ma Y Goldstein MR Davis RB Yeh GY. Profile of subjective-objective sleep discrepancy in patients with insomnia and sleep apnea. J Clin Sleep Med. (2021) 17:2155–63. doi: 10.5664/jcsm.9348, 34666882 PMC8636379

[8] Espie CA Broomfield NM MacMahon KM Macphee LM Taylor LM. The attention-intention-effort pathway in the development of psychophysiologic insomnia: a theoretical review. Sleep Med Rev. (2006) 10:215–45. doi: 10.1016/j.smrv.2006.03.002, 16809056

[9] Wei H Zhu J Lei F Luo L Zhang Y Ren R . Clinical phenotypes of obstructive sleep apnea: a cluster analysis based on sleep perception and sleep quality. Sleep Breath. (2023) 27:1829–37. doi: 10.1007/s11325-023-02786-4, 36853471 PMC10539408

[10] Martín-Montero A Vaquerizo-Villar F García-Vicente C Gutiérrez-Tobal GC Penzel T Hornero R. Heart rate variability analysis in comorbid insomnia and sleep apnea (COMISA). Sci Rep. (2025) 15:17574. doi: 10.1038/s41598-025-02541-7, 40399494 PMC12095586

[11] Shi Y Feng X Hao F Nie Y Zhang Y Chen Z . Clinical subtypes of co-morbid insomnia and obstructive sleep apnea (COMISA): results of a cluster analysis. Respir Res. (2026) 27:136. doi: 10.1186/s12931-026-03577-7, 41680824 PMC12998145

[12] Sweetman A Lack L McEvoy RD Smith S Eckert DJ Osman A . Bi-directional relationships between co-morbid insomnia and sleep apnea (COMISA). Sleep Med Rev. (2021) 60:101519. doi: 10.1016/j.smrv.2021.101519, 34229295

[13] Chinese Medical Association, Chinese Medical Association Publishing House, General Practice Branch of Chinese Medical Association, . Guideline for primary care of adult obstructive sleep apnea (practice version, 2018). Chin J Gen Pract. (2019) 18:30–5. doi: 10.3760/cma.j.issn.1671-7368.2019.01.008

[14] Zhou LQ Zhang JN Peng Y . Reliability, validity and related factors of the modified Epworth sleepiness scale. Chin J Health Stat. (2006) 3:218–20. doi: 10.3969/j.issn.1002-3674.2006.03.008

[15] Ilic I Babic G Dimitrijevic A Ilic M Sipetic Grujicic S. Internal consistency and validity of the hospital anxiety and depression scale (HADS) in women with abnormal pap smear in Serbia. Women Health. (2021) 61:363–71. doi: 10.1080/03630242.2021.1893244, 33641629

[16] Liu XC Tang MQ Hu L . Reliability and validity of the Pittsburgh sleep quality index. Chin J Psychiatry. (1996) 29:103–107. doi: 10.3760/cma.j.issn.1006-7884.1996.02.111

[17] Wang W Wang LN. Application of the Montreal cognitive assessment in screening patients with mild cognitive impairment. Chin J Intern Med. (2007) 46:414–6. doi: 10.3760/j.issn:0578-1426.2007.05.031

[18] Tian M Song YL Zhang XQ . Latent profile analysis of sleep subtypes and associated factors in older adults with subjective cognitive decline. Chin Gen Pract. (2023) 26:3297–302. doi: 10.12114/j.issn.1007-9572.2023.0096

[19] Zhang XQ Zeng H. Effect of test content adjustment of the Beijing version of the Montreal cognitive assessment on its test performance. Chin Nurs Res. (2013) 27:1268–9. doi: 10.3969/j.issn.1009-6493.2013.13.053

[20] Chen PF Liu YX Wang TZ . Intervention effect of online brief behavioral therapy for insomnia on insomnia disorder. Chin Gen Pract. (2024) 27:4370–5. doi: 10.12114/j.issn.1007-9572.2024.0052

[21] Berry RB Budhiraja R Gottlieb DJ Gozal D Iber C Kapur VK . Rules for scoring respiratory events in sleep: update of the 2007 AASM manual for the scoring of sleep and associated events. Deliberations of the sleep apnea definitions task force of the American Academy of sleep medicine. J Clin Sleep Med. (2012) 8:597–619. doi: 10.5664/jcsm.2172, 23066376 PMC3459210

[22] Mölle M Bergmann TO Marshall L Born J. Fast and slow spindles during the sleep slow oscillation: disparate coalescence and engagement in memory processing. Sleep. (2011) 34:1411–21. doi: 10.5665/sleep.1290, 21966073 PMC3174843

[23] Bianchi MT Wang W Klerman EB. Sleep misperception in healthy adults: implications for insomnia diagnosis. J Clin Sleep Med. (2012) 8:547–54. doi: 10.5664/jcsm.2154, 23066367 PMC3459201

[24] Naito K Iwamoto K Miyata S Okada I Kawai K Fujimoto A . Effect of lemborexant on sleep-wake state discrepancy in participants with insomnia disorder. Psychopharmacology. (2025) 243:133–43. doi: 10.1007/s00213-025-06845-4, 40553164 PMC12881114

[25] Lee SA Im K Yang HR. Factors associated with sleep state misperception in patients with obstructive sleep apnea. Sleep Breath. (2022) 26:1921–30. doi: 10.1007/s11325-021-02543-5, 35028861

[26] Nam H Lim JS Kim JS Lee KJ Koo DL Lee C. Sleep perception in obstructive sleep apnea: a study using polysomnography and the multiple sleep latency test. J Clin Neurol. (2016) 12:230–5. doi: 10.3988/jcn.2016.12.2.230, 27074296 PMC4828571

[27] Lee SA Im K Yang HR. Effects of continuous positive airway pressure on sleep state misperception in patients with obstructive sleep apnea. J Korean Med Sci. (2023) 38:e54. doi: 10.3346/jkms.2023.38.e54, 36852850 PMC9970791

[28] Kuhn J Schiphorst LRB Wulterkens BM Asin J Duis N Overeem S . Multi-night home assessment of total sleep time misperception in obstructive sleep apnea with and without insomnia symptoms. Clocks Sleep. (2024) 6:777–88. doi: 10.3390/clockssleep6040050, 39727626 PMC11674459

[29] Choi SJ Suh S Ong J Joo EY. Sleep misperception in chronic insomnia patients with obstructive sleep apnea syndrome: implications for clinical assessment. J Clin Sleep Med. (2016) 12:1517–25. doi: 10.5664/jcsm.6280, 27568893 PMC5078707

[30] Zhao W Du J Liu Y . Longitudinal changes of obstructive sleep apnea and its impact on cardiovascular outcomes: results from the sleep heart health study. Pulmonology (Madr). (2026) 32:2640670. doi: 10.1080/25310429.2026.2640670, 41906805

[31] Soh N Rainey-Smith SR Doecke JD Canovas R Bucks RS Ree M . Sleep discrepancy and cognitive function in community-dwelling older adults. J Sleep Res. (2025) 34:e14288. doi: 10.1111/jsr.14288, 39054858 PMC11744247

[32] Liang Y Zhao X Zhang C Liu G Lu B Han L . Sleep misperception and associated factors in patients with anxiety-related disorders and complaint of insomnia: a retrospective study. Front Neurol. (2022) 13:836949. doi: 10.3389/fneur.2022.836949 35463154 PMC9021819

[33] Nakagawa Y Yamada S. Novel hypothesis and therapeutic interventions for irritable bowel syndrome: interplay between metal dyshomeostasis, gastrointestinal dysfunction, and neuropsychiatric symptoms. Mol Cell Biochem. (2025) 480:2661–76. doi: 10.1007/s11010-024-05153-3, 39503802

[34] Lin B Wang D Schroevers MJ Huang Y Chong ZY Liu X . Prefrontal activation and inhibitory performance in the association between anxiety and sleep quality: an fNIRS investigation. J Affect Disord. (2026) 401:121245. doi: 10.1016/j.jad.2026.121245, 41605351

[35] Fabbri M Simione L Catalano L Mirolli M Martoni M. Attentional bias for sleep-related words as a function of severity of insomnia symptoms. Brain Sci. (2022) 13:50. doi: 10.3390/brainsci13010050, 36672032 PMC9856532

[36] Xiao S Shi L Zhang J Li X Lin H Xue Y . The role of anxiety and depressive symptoms in mediating the relationship between subjective sleep quality and cognitive function among older adults in China. J Affect Disord. (2023) 325:640–6. doi: 10.1016/j.jad.2023.01.048, 36657496

[37] Aksakal E Kaya F Aksakal A. Sleep quality and general distress in individuals with obstructive sleep apnoea syndrome: the mediating role of emotion regulation. Aust Psychol. (2026) 61:211–25. doi: 10.1080/00050067.2025.2567678

[38] Aksakal A Aksakal E Kaya F Kerget B Afşin DE Araz Ö. Psychological resilience as a mediator between sleep quality and mental well-being in patients with obstructive sleep apnea syndrome. BMC Psychol. (2025) 13:979. doi: 10.1186/s40359-025-03357-w, 40877995 PMC12395750

